# Electrochemical Degradation of Venlafaxine on Platinum Electrodes: Identification of Transformation Products by LC-MS/MS and In Silico Ecotoxicity Assessment

**DOI:** 10.3390/molecules30091881

**Published:** 2025-04-23

**Authors:** Angelica R. Zizzamia, Veronica Pasquariello, Filomena Lelario, Carmen Tesoro, Rosanna Ciriello

**Affiliations:** Department of Basic and Applied Sciences, University of Basilicata, 85100 Potenza, Italy; angelicarebecca.zizzamia@unibas.it (A.R.Z.); veronica.pasquariello@studenti.unibas.it (V.P.); carmen.tesoro@unibas.it (C.T.)

**Keywords:** electrochemical degradation, venlafaxine, platinum plate, LC-MS, degradation intermediates, in silico toxicity

## Abstract

Antidepressants are emerging contaminants that have raised global concern due to their abuse. Venlafaxine (VFX), a serotonin and norepinephrine reuptake inhibitor, can cause adverse and potentially toxic effects on aquatic organisms. Electrochemical advanced oxidation processes (EAOPs) are gaining attention as promising degradation techniques for a variety of drugs. EAOP methods proposed for VFX degradation mainly utilize boron-doped diamond (BDD) electrodes, characterized by low background current and high oxygen overpotential. However, challenges arise, including delamination from the substrate, difficulties in scaling up, and limited service life. In this study, platinum was employed as an anode for the galvanostatic degradation of VFX, due to its stability and well-established surface cleaning procedure, which ensured high reproducibility. A 0.1 M Na_2_SO_4_ solution at pH 9 was used as the supporting electrolyte, and a current density of 25 mA/cm^2^ was applied. After 7 h, a degradation efficiency of 94% was achieved for a 25 ppm VFX solution. The hydroxyl and sulfate radicals generated in the electrochemical system were the active species responsible for VFX degradation, which followed a first-order kinetic model with a rate constant of 0.0084 min^−1^. The main degradation intermediates were identified through LC-MS, including two isomers with a nominal *m*/*z* of 276 and three isomers with a nominal *m*/*z* of 294. The toxicity of the VFX degradation products was assessed by an in silico prediction model. This evaluation confirmed the sustainability of the developed method.

## 1. Introduction

Emerging contaminants (ECs) are synthetic or naturally occurring compounds not routinely monitored but potentially harmful to ecosystems and human health [1]. Among them, pharmaceuticals have been included in the list of emerging contaminants from UNESCO [2]. Particularly, antidepressants have been frequently detected in surface and groundwater at concentrations ranging from ng L^−1^ to mg L^−1^ [3]. Venlafaxine, a serotonin and norepinephrine reuptake inhibitor, is one of the most widely consumed antidepressants and has been found in various aquatic environments worldwide [4].

Due to its low absorption in humans (approximately 87% of the total dose), VFX is predominantly excreted and enters water systems via domestic wastewater, hospital discharges, and industrial effluents. Its persistence and poor biodegradability raise environmental concerns, as it has been shown to affect aquatic organisms, including neurobehavioral and reproductive disruptions [5]. Accordingly, VFX has been listed in the third EU Watch List of emerging pollutants [6].

Conventional wastewater treatment plants (WWTPs) have proven to be inadequate for the complete removal of venlafaxine, with efficiencies ranging from 7.7% to 56% [7]. In contrast, advanced oxidation processes (AOPs) represent a promising alternative to traditional water treatment methods. These processes employ various reaction systems to generate highly reactive hydroxyl radicals capable of attacking a broad range of organic contaminants [8], including VFX.

Consequently, several AOPs have been investigated for VFX degradation [9,10,11,12]. However, despite the high reactivity, methods like ozonation often suffer from the practical constraints of in situ generation and substantial operational costs [12]. Similarly, TiO_2_ photocatalysis has strong oxidation capabilities and low toxicity [10,11], but it presents challenges related to catalyst recovery and efficient light utilization. Although these AOPs have shown improved removal of certain persistent compounds, including VFX, these limitations highlight the need for innovative solutions.

Among AOPs, electrochemical-based advanced oxidation processes are gaining increasing interest due to their simplicity, cost-effectiveness, safety, and environmental sustainability. Indeed, EAOPs rely on the electrochemical generation of strong oxidants without the need for harmful chemical reagents [13]. Electrochemical anodic oxidation can proceed via direct or indirect mechanisms. In the direct pathway, contaminants are oxidized directly at the anode surface. In the indirect pathway, reactive radical species generated electrochemically mediate the oxidation. The simplest indirect route involves the formation of hydroxyl radicals (^•^OH) via water electrolysis; these radicals subsequently cause the mineralization of organic pollutants. Other reactive radicals can also be electrogenerated from added precursors such as chloride, sulfate, and persulfate ions. Several factors can influence the nature and amount of reactive species formed during the EAOP process, such as the electrolyte composition, applied potential or current, and most critically, the anode material used [14].

As far as VFX is concerned, there are only a few studies on its electrochemical degradation, all based on the employment of BDD electrodes as anode [15]. A comparison between electrochemical and photoinduced degradation showed both AOPs to be effective, though photoinduced degradation occurred much faster. The degradation mechanisms were investigated, and the results showed that photoinduced degradation primarily followed the indirect pathway mediated by ^•^OH radicals, while electrochemical oxidation involved both direct and indirect pathways [16]. The effect of other radical species on VFX degradation was also examined. The presence of chloride ions in the electrolyte solution significantly accelerated the decomposition, and 98.5% of VFX (25 mg/L) was removed within 5 min [17]. Cl^•^ radicals contributed to the opening of the benzene ring, which facilitated rapid degradation. Conversely, the addition of tert-butanol, a known ^•^OH radical quencher, reduced the degradation rate. This outcome confirmed the key role of ^•^OH radicals in the process. Additionally, bicarbonate and phosphate anions acted as radical scavengers and inhibited VFX degradation [18].

Boron-doped diamond anodes possess valuable physicochemical properties such as wide potential window, low background current, high oxygen overpotential, and corrosion stability [19]. As inert electrodes, they allow weak adsorption of electrochemically generated hydroxyl radicals, and thus enhance their availability for reactions with contaminants in solution. These characteristics support their extensive use in the oxidation and mineralization of organic compounds. However, several limitations must be addressed [20]. BDD films are typically deposited on substrates such as silicon, tungsten, molybdenum, titanium, niobium, or tantalum using chemical vapor deposition (CVD) techniques. Mechanical cleaning methods may damage the electrode surface and should be avoided. Electrochemical cleaning protocols are preferred, though they significantly affect the electrode’s properties and lack standardization due to morphological variability among BDD electrodes [21]. Additionally, electrochemical pre-treatment often requires high current densities or potentials, which may damage adhesive materials used to attach the BDD layer and thus compromise electrode performance and reproducibility.

BDD electrodes are susceptible to delamination from the substrate. To select the optimal substrate, it is crucial to assess the compatibility of thermal expansion coefficients. A substrate with a significantly different coefficient can lead to structural defects, which allow electrolyte penetration and cause substrate corrosion [22]. Silicon and tantalum are the most compatible materials; however, silicon exhibits fragility and low electrical conductivity, while tantalum is expensive. Other materials like nickel, tungsten, and titanium show good electrochemical performance but suffer from delamination issues. Additionally, silicon, tantalum, niobium, and tungsten are not suitable for industrial-scale applications [23]. As a result, the exploitation of other electrode materials as anodes would undoubtedly be beneficial to advance EAOP technology.

This study addresses the limitations of existing EAOP methods for venlafaxine degradation. It presents a comparative analysis of platinum and glassy carbon anodes, with a focus on elucidating the critical difference between direct and indirect oxidation mechanisms. Despite the absence of direct VFX oxidation on platinum evidenced from cyclic voltammetry experiments, this material allowed us to achieve a higher degradation efficiency through indirect oxidation, a crucial insight often overlooked in studies primarily focused on BDD electrodes. Degradation through indirect oxidation was the mechanism prevalent even on electrodes capable of direct VFX oxidation, like glassy carbon. Understanding these electrode-specific mechanisms is paramount for rational EAOP design.

In this work, platinum is therefore proposed for the first time as a viable alternative to BDD anode in the electrolytic degradation of VFX. Notably, platinum offers the advantage of a well-established cleaning procedure, ensuring high reproducibility of the surface state, and it is stable and usable over an extended period [24]. The electrochemical degradation process was optimized under environmentally relevant conditions, i.e., near neutral pH of supporting electrolyte and moderate current density, specifically avoiding the use of chloride-containing electrolytes to prevent the formation of harmful disinfection by-products, which is a major concern in water treatment. It is worth noting that many studies achieved high degradation efficiency but used chloride-based electrolytes. This choice limits their practical applicability.

A comprehensive identification of transformation products is provided through LC-MS/MS. Detailed fragmentation studies were conducted to differentiate between isomers. This analysis provided valuable insights into the degradation pathways of venlafaxine. Furthermore, this investigation was coupled with an in silico ecotoxicity assessment using the ECOSAR (Ecological Structure Activity Relationships, V2.2) predictive model which demonstrated the sustainability of the developed degradation technique. This approach, consistent with established hazard prediction methodologies [16], offers a more complete evaluation of the environmental impact of the treatment process compared to studies solely focused on parent compound removal.

## 2. Results

### 2.1. Evaluation of Anode Material for the Electrochemical Degradation of VFX

The anode material is a key parameter influencing the electrochemical degradation efficiency of emerging contaminants. VFX is an electroactive compound that, therefore, can undergo degradation through direct anodic oxidation. To identify the electrode able to provide the highest degradation efficiency, a preliminary investigation on the electrochemical behavior of VFX on platinum and glassy carbon electrodes was carried out.

Glassy carbon (GC) is a structurally ordered, sp^2^-hybridized, non-graphitizable carbon with low permeability, high thermal resistance, and good electrical conductivity. Its fullerene-related structure, formed by curved graphene layers enclosing closed pores, provides exceptional chemical and mechanical stability [25].

**Figure 1:**
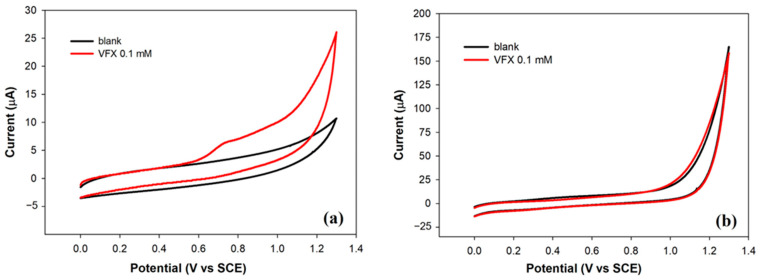
Cyclic voltammograms acquired in phosphate buffer solution (0.1 M, pH 7) at a bare platinum electrode (**a**) and at a bare glassy carbon electrode (**b**) in the absence (black curves) and in the presence of VFX at a concentration of 0.1 mM (red curves). Scan rate: 50 mV/s.

To investigate the electrochemical behavior of VFX on this electrode, the voltammetric profile of a 0.1 mM solution in phosphate buffer at pH 7 was recorded (Figure 1a). During the anodic scan, a current peak was observed at a potential of about 0.74 V, attributable to the irreversible oxidation of VFX, as no cathodic peak was present in the backward scan. The flat voltammetric profile acquired in the pure electrolyte further supported this attribution.

A similar investigation was carried out on the Pt electrode. Platinum anodes are electrode materials typically used for the oxidation of organic pollutants, due to their good electrical conductivity and chemical stability [26]. The voltammetric profile of VFX on Pt, shown in Figure 1b, closely resembled that acquired in the pure electrolyte solution. The absence of the characteristic oxidation peak suggested that venlafaxine is not electroactive on platinum electrodes, at least within the potential range explored.

In order to gain a deeper understanding of the electrochemical behavior of VFX on the GC electrode, the influence of scan rate on the voltammetric profile was investigated. Useful information involving electrochemical mechanisms generally can be acquired from the relationship between peak current and scan rate which allows us to assess whether the electrochemical process is under diffusion or adsorption control. Peak currents evaluated from the voltammograms recorded in the range of 5–150 mV/s displayed a linear relationship with the scan rate (r^2^ = 0.9956), characteristic of adsorption-controlled electrochemical processes [27]. It is reasonable to assume that the adsorption of VFX is driven by π–π interactions with glassy carbon. Additionally, a shift of the peak potential toward more positive values with increasing scan rate was observed which further confirmed the irreversibility of the oxidation process. This behavior may explain the lack of electroactivity on platinum: the poor affinity for this material prevents VFX from adsorbing onto the electrode surface, which is essential for oxidation to occur.

As previously discussed, drugs can be electrochemically degraded either through direct oxidation or by interaction with reactive radical species produced at the electrode. Both glassy carbon and platinum plates were then tested as anodes for the electrochemical degradation of venlafaxine, regardless of the electroactivity exhibited by VFX. A graphite plate was used as a cathode in all the experiments.

The galvanostatic electrochemical degradation was first carried out by applying a current density of 10 mA/cm^2^ for 60 min, employing a 25 ppm solution of VFX in phosphate buffer 0.1 M at pH 7, kept under stirring during the experiment. An estimation of the degradation extent was driven from the current profiles of the VFX solution acquired by differential pulse voltammetry (DPV) on a conventional glassy carbon electrode and on a platinum electrode before and after the electrolysis (Figure S1). The peak current abatement was 25% and 40% when glassy carbon and platinum plates were used as anodes during the electrolysis, respectively. The apparent discrepancy between CV and electrolysis findings can be explained by the dominance of an indirect oxidation pathway in the degradation process. While voltammetric analysis showed no direct electron transfer from VFX to the platinum electrode, electrolysis revealed significant degradation efficiency under the applied current. This was due to the continuous electrochemical generation of hydroxyl radicals at the platinum surface, which then oxidized VFX.

To further support the hypothesis of the indirect oxidation pathway, the degradation experiment on the glassy carbon plate was repeated by adding 200 mM ethanol to the electrolyte solution. Ethanol is commonly used in the literature as a scavenger of ^•^OH radicals [28]. Free radical quenching experiments help to identify the type of active species and assess their role in anodic oxidation processes. These radical scavengers selectively interact with active species and compete with pollutants because of their high reactivity.

From the DPV profiles obtained before and after electrolysis in the presence of ethanol (Figure S2), a current decrease of only 8% was observed, significantly lower than the decrease found in the absence of ethanol. This suggests that even on glassy carbon, the drug degradation primarily occurred through indirect mechanisms mediated by ^•^OH radicals. While indirect evidence from radical scavenging experiments indicated the involvement of hydroxyl radicals, future studies employing electron paramagnetic resonance (EPR) spectroscopy could provide direct confirmation of their formation during the electrochemical process. Since venlafaxine degraded more on platinum electrodes than on glassy carbons, platinum was selected as the anode for the subsequent electrochemical degradation experiments.

### 2.2. Optimization of the Degradation Conditions

The efficiency of EAOP is highly dependent on the experimental conditions used to carry out electrolysis, which were optimized in this study to ensure maximum removal of the drug and its transformation products. The change in VFX concentration during electrochemical degradation experiments was monitored by taking 1 mL aliquots from the electrolyte solutions at fixed time intervals and analyzing them by HPLC-UV. Methanol was added to each of the analyzed solutions due to its compatibility with the mobile phase and its effectiveness in quenching residual radicals during sample storage. It should be emphasized that ethanol was employed in the mechanistic studies with glassy carbon electrodes because of its well-established selectivity for hydroxyl radicals. This choice enabled a more specific assessment of their role in the degradation pathway.

In electrochemical degradation, a supporting electrolyte is added to improve solution conductivity and facilitate the generation of strong oxidants. Sodium sulfate is considered an “active” electrolyte as it allows the formation of sulfate radicals (SO_4_^•−^) through various pathways, primarily via reaction with hydroxyl radicals (^•^OH) produced during water electrolysis [29]. The rate of SO_4_^•−^ formation is thus limited by the availability of ^•^OH radicals. A higher sulfate concentration does not increase the production of active species or enhance pollutant removal. Moreover, excess sulfate may also lead to anode fouling, which impairs electrolytic performance. Therefore, a standard concentration of 0.1 M is commonly used. The starting experimental conditions for VFX degradation were then based on the employment of 0.1 M sodium sulfate at pH 7 as a supporting electrolyte.

Advanced electrochemical oxidation processes generally operate in galvanostatic mode. Current density is a key parameter since it controls the amount of oxidizing species produced. Theoretically, an increase in current density should promote the generation of free radicals on the anode surface. As a result, the time required for contaminant degradation would be reduced. However, there is a threshold value beyond which the high voltage generated can trigger undesirable reactions on the anode, such as oxygen evolution. These reactions result in the formation of non-oxidizing species that do not contribute to the further degradation of VFX.

To evaluate this threshold value, degradation experiments were repeated by applying a current density of 5, 10, and 25 mA/cm^2^, using a solution of 25 ppm venlafaxine in 0.1 M Na_2_SO_4_ at pH 7.

**Figure 2:**
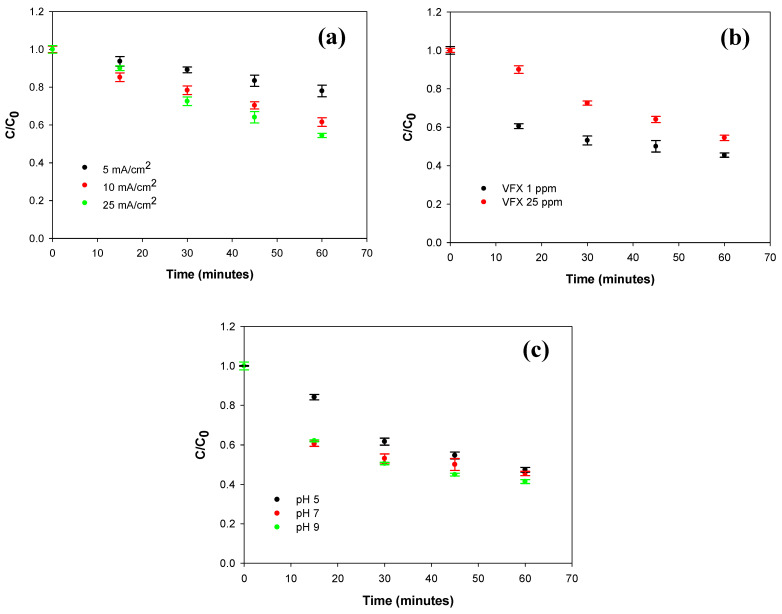
(**a**) Normalized concentration–time curves for the electrochemical degradation of a 25 ppm solution of VFX in Na_2_SO_4_ 0.1 M at pH 7 realized by applying a current density of 5 mA/cm^2^ (black circles), 10 mA/cm^2^ (red circles), and 25 mA/cm^2^ (green circles); (**b**) Normalized concentration–time curves for the electrochemical degradation of a 25 ppm (red circles) and 1 ppm (black circles) solution of VFX in Na_2_SO_4_ 0.1 M at pH 7 realized by applying a current density of 25 mA/cm^2^; (**c**) Normalized concentration–time curves for the electrochemical degradation of a 1 ppm solution of VFX in Na_2_SO_4_ 0.1 M at pH 5 (black circles), 7 (red circles), and 9 (green circles) realized by applying a current density of 25 mA/cm^2^. Data points represent the mean ± SD of n = 3 independent experiments.

In Figure 2a, the degradation curves obtained for each experiment by LC-UV are reported as C/C_0_ ratio versus time, where C represents the venlafaxine concentration at a given time during electrolysis, and C_0_ is the initial concentration. After 60 min, the highest degradation percentage (45.5%) was observed in the experiment conducted at 25 mA/cm^2^. The application of current densities of 5 and 10 mA/cm^2^ resulted in lower degradation efficiencies of 22% and 38.5%, respectively. No significant improvements were observed when the current density was increased further. Based on these experimental results, a current density of 25 mA/cm^2^ was selected for the subsequent experiments.

The influence of VFX concentration on the degradation efficiency was also investigated. Two different initial concentrations were used, 25 ppm and 1 ppm, while the other experimental conditions were kept at their previously optimized values. The relevant degradation curves are depicted in Figure 2b. At the 60-minute mark, the removal efficiencies were 45.5% for 25 ppm and 54.5% for 1 ppm. The lower efficiency at 25 ppm can be explained considering that, under fixed operating conditions, the amount of reactive species, i.e., free radicals, remains nearly constant, while the concentration of the organic compound is higher and therefore requires a longer time to achieve the same level of degradation [30]. Furthermore, the concentration of transformation products is also increased. As a result, competition between VFX and its intermediates for reaction with radical species becomes more intense.

The pH of the electrolyte solution is a key factor in electrochemical degradation, as wastewater often exhibits different pH values. Studies on pH effects have yielded inconsistent results, which highlight its complex role [31]. pH can affect both pollutant behavior and the generation of reactive species, but can also promote the formation of species that quench their activity. In this study, the degradation of VFX over time was monitored at three different pH values of the electrolyte solution: 5, 7, and 9. The experiment was conducted using a venlafaxine solution at 1 ppm, in 0.1 M Na_2_SO_4_, a current density of 25 mA/cm^2^ was applied. The degradation curves are illustrated in Figure 2c. After 60 min of electrolysis, a slight improvement in degradation efficiency was observed at pH 9 (59%), probably due to an increased formation of ^•^OH radicals favored by the higher concentration of OH^−^ ions. While higher pH values (e.g., [10,11]) might further enhance degradation, we focused on conditions that are typical of wastewater pH ranges. Future studies could explore the impact of these higher pH values on degradation efficiency.

**Figure 3:**
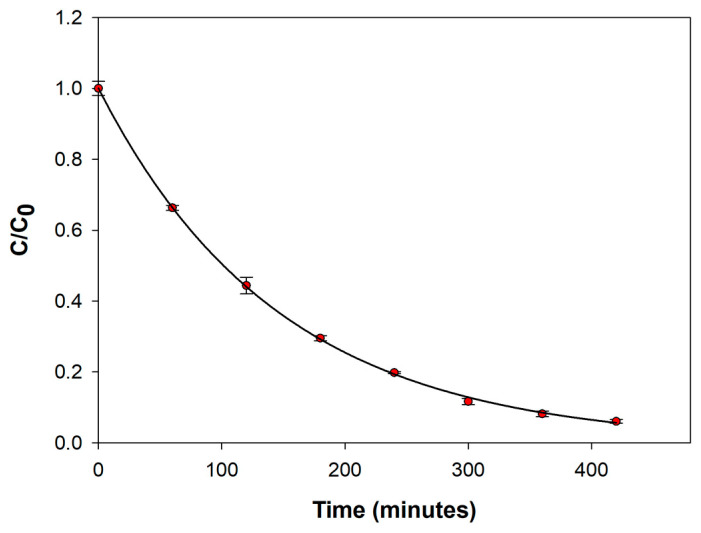
Normalized concentration–time curve for the electrochemical degradation of a 25 ppm solution of VFX in Na_2_SO_4_ 0.1 M at pH 9 realized by applying a current density of 25 mA/cm^2^. The analysis was performed in triplicate, and the results are expressed as mean ± standard deviation (SD).

The degradation curve for longer electrolysis time was derived by employing the electrolysis conditions optimized. Particularly, a venlafaxine concentration of 25 ppm was used in order to assure an appreciable concentration of the degradation products, which allowed for their identification by LC-MS. The degradation curve obtained after 7 h (420 min) of electrolysis is depicted in Figure 3. The experiment was repeated three times, and remarkable precision was achieved due to the reproducibility of the platinum surface state (coefficients of variation ranging from 1.04 to 9.77%). A high venlafaxine degradation efficiency of 94% was achieved. The degradation process followed a first-order kinetic model with a rate constant and a half time of 0.0084 min^−1^ and 82.52 min, respectively.

It is worth noting that shorter degradation times with high degradation efficiency are reported and achieved by adding chloride ions to the electrolyte solution [17]. Additionally, the working conditions in those studies involved acidic pH [16] and high current densities [18]. In contrast, the method proposed here operates under milder conditions and, importantly, does not rely on accelerants like chloride, which inevitably lead to the formation of hazardous by-products.

### 2.3. Structural Elucidation of Venlafaxine and Degradation Products by LC-ESI-LIT-MS^n^ and LC-ESI-Orbitrap-MS

LC-DAD experiments were carried out to monitor the degradation pathway of venlafaxine over time under varying electrolysis conditions. Optimal degradation was observed under the following experimental parameters: pH 9; 0.1 M Na_2_SO_4_ solution; current density of 25 mA/cm^2^. Due to the limitations of retention time and UV spectra, further analytical techniques were required for the definitive identification of constituents within complex matrices, particularly for unknown compounds. Therefore, an LC-MS study of the drug and its degradation products was performed using low and high resolutions. Electrochemical degradation was achieved under optimized experimental conditions using a drug concentration of 25 ppm. In addition, CID-MS^n^ experiments were conducted to elucidate the transformation products’ fragmentation pathways and gain further structural information.

#### 2.3.1. LC-ESI-Orbitrap-MS Studies of Venlafaxine and Its Degradation Products

**Figure 4:**
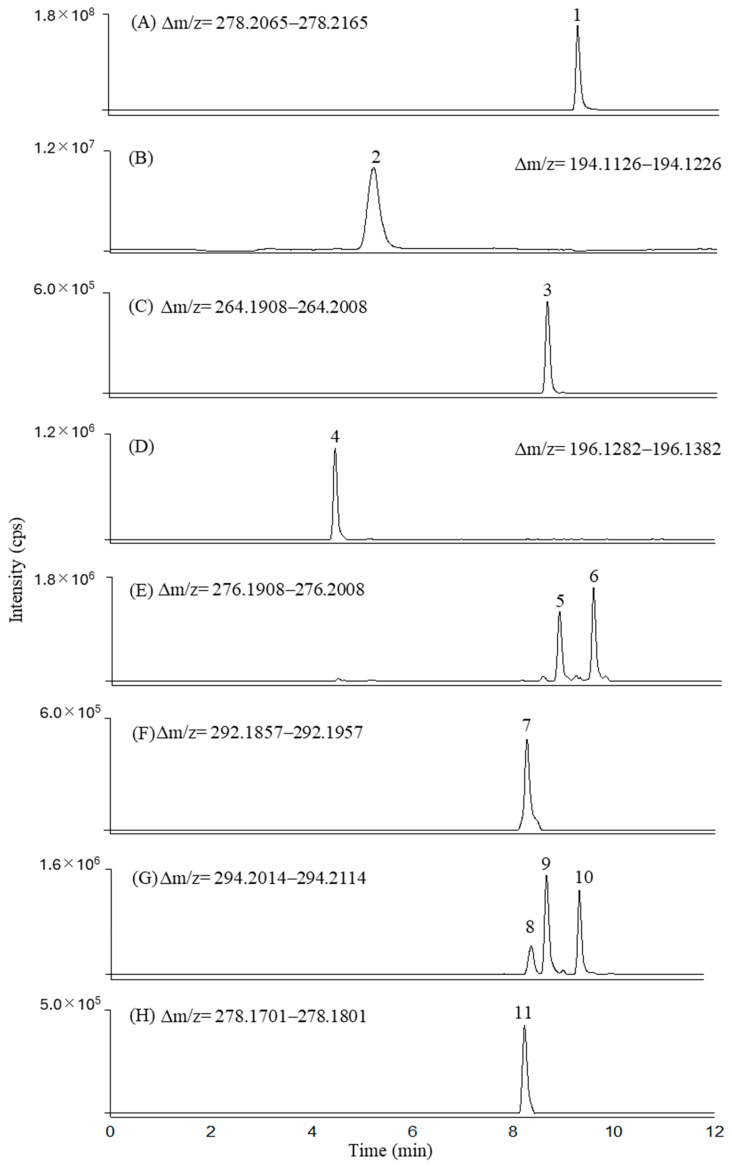
Extracted ion chromatograms (XICs) acquired for the following compounds: venlafaxine (**A**), V194 (**B**), V264 (N-desmethylvenlafaxine, (**C**), V196 (**D**), V276a (RT 8.80 min, peak 5) and V276b (RT 9.48 min, peak 6) (**E**), V292 (**F**), V294a (RT 8.49 min, peak 8), V294b (RT 8.83 min, peak 9), V294c (RT 9.47 min, peak 10) (**G**), and V278 (peak 11) (**H**).

The composition of VFX degradation mixtures was elucidated using liquid chromatography–electrospray ionization–orbitrap mass spectrometry (LC-ESI-Orbitrap-MS). While total ion current chromatograms (TICs) were initially acquired, their interpretative limitations, due to the overlap of coeluting ions, necessitated the use of extracted ion chromatograms (XICs). XICs selectively displayed ion intensities within a narrow mass-to-charge ratio window of [M+H]^+^ ± 5.0 mDa, which enabled the identification of individual compounds, including low-abundance transformation products, and minimized signal interferences. This method was applied to VFX degradation mixtures at various times and enabled product separation. Figure 4 shows the XICs of protonated venlafaxine and its transformation products after 100 min of electrochemical degradation at pH 9, a mix with significant product concentrations.

All chromatographic peaks were identified through accurate mass measurements, which enabled the determination of molecular formulas and the interpretation of MS^n^ data in combination with literature comparisons. Fragmentation studies based on collision-induced dissociation (CID) were performed for each identified product, which allowed us to confirm their identity based on the analysis of their respective fragmentation pathways. Chromatographic and mass spectrometry data for the compounds identified in the degradation mixture are summarized in Table 1. This table includes retention times, molecular formulas, exact and accurate *m*/*z* values, associated mass errors, and main fragments of the [M+H]^+^ ions for each compound. Notably, the mass errors for the accurate mass values were consistently below 1.6 ppm, demonstrating good mass accuracy.

**Scheme 1:**
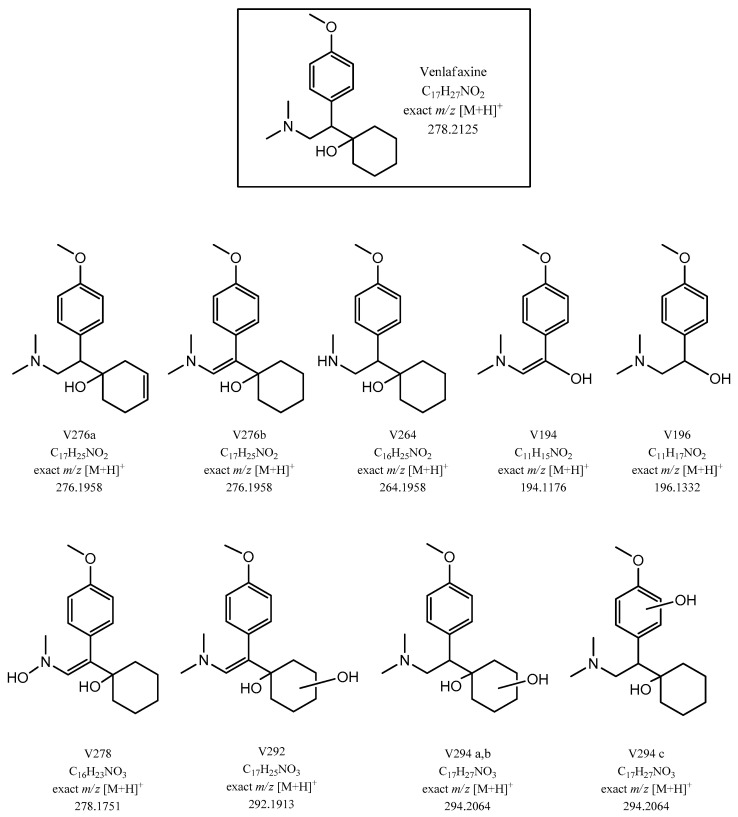
Proposed chemical structures, molecular formulas, and exact *m*/*z* values of venlafaxine transformation products.

MS^n^ data obtained using a linear ion trap (LIT) analyzer with CID confirmed the identity of the compounds and facilitated unambiguous structural elucidation. These results align with literature reports concerning venlafaxine degradation, specifically photochemical degradation processes, and electrochemical degradation using a boron-doped diamond electrode [16]. As depicted in Figure 4 and detailed in Table 1, ten main transformation intermediates were identified. Among these were two isomers with an exact *m*/*z* ratio of 276.1958 (V276a and V276b, Figure 4E) and three isomers with an exact *m*/*z* ratio of 294.2064 (V294a, V294b, and V294c, Figure 4G). Accurate mass measurements alone could not differentiate between these isomers, as they provide only molecular formula information. Therefore, fragmentation studies were performed, by isolating chromatographic peaks that corresponded to each identified product. The chemical structures proposed for the transformation products are shown in Scheme 1.

#### 2.3.2. LC-ESI-CID-MS^n^ Studies of VFX Degradation Products

**Figure 5:**
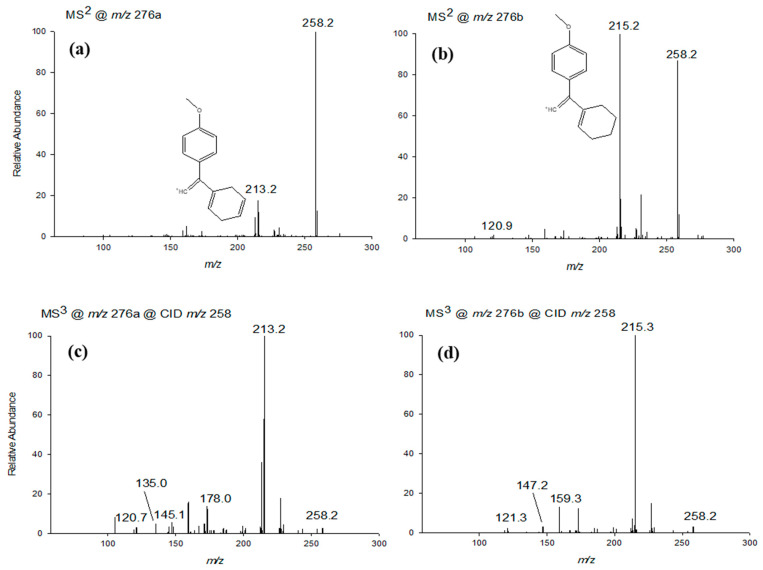
CID-MS^n^ spectra (**a**–**d**) in positive mode of the [M+H]^+^ ion of the two isomers at nominal *m*/*z* 276 generated by applying relative collision energies between 30% and 35%.

As previously mentioned, the degradation products of venlafaxine include two isomers with an exact *m*/*z* value of 276.1958 (V276a and V276b, C_17_H_25_NO_2_^+^), eluting at 8.80 and 9.48 min, respectively. Their CID-MS^n^ spectra are presented in Figure 5. The 2 Da mass difference from *m*/*z* 278 (VFX) was consistent with the formation of a double bond in either the cyclohexane ring (isomer a) or the carbon chain (isomer b).

The MS^2^ spectrum of isomer V276a (Figure 5a) demonstrated that fragmentation of the molecular ion yielded two fragment ions at *m*/*z* 258 and 213, corresponding to the structures C_17_H_23_NO^+^ and C_15_H_17_O^+^, respectively. The ion at *m*/*z* 258 derived from V276a through dehydration and subsequent formation of a second double bond in the cyclohexane ring. The ion at *m*/*z* 213 resulted from the *m*/*z* 258 fragment through the loss of the dimethylamine group -NC_2_H_7_ (−45 Da).

The MS^2^ spectrum of isomer V276b (Figure 5b) also showed that fragmentation of the molecular ion produced fragment ions at *m*/*z* 258 and 215, corresponding to the molecular formulas C_17_H_23_NO^+^ and C_15_H_19_O^+^, respectively. Similar to isomer V276a, the ion at *m*/*z* 258 was generated by dehydration of V276b, accompanied by the introduction of a conjugated double bond and the transformation of cyclohexane to cyclohexene. The fragment at *m*/*z* 215 was generated from *m*/*z* 258 by the loss of the -NC_2_H_5_ amino group (−43 Da).

The chemical structures of the fragments at *m*/*z* 213 and 215, depicted in the MS^2^ spectra of the two isomers (Figure 5a and Figure 5b, respectively), confirmed the presence of a double bond in different regions of the molecule.

The MS^3^ spectrum of V276a (Figure 5c), obtained from the fragmentation of the *m*/*z* 258 ion, revealed the presence of five fragment ions at nominal *m*/*z* 213, 121, 135, 145, and 178, with *m*/*z* 213 showing the highest intensity. The MS^3^ spectrum of V276b (Figure 5d), also derived from the fragmentation of *m*/*z* 258, displayed four fragment ions (previously observed) at *m*/*z* 215 (the most intense), 121, 147, and 159. The fragments at *m*/*z* 147 (C_10_H_11_O^+^) and 215 (C_15_H_19_O^+^) were characteristic of venlafaxine MS^n^ spectra (see Table 1). The peaks at *m*/*z* 135 and 178 corresponded to the fragments C_9_H_11_O^+^ and C_11_H_16_NO^+^, respectively. The C_11_H_16_NO^+^ fragment (*m*/*z* 178) was due to the loss of the hydroxy-substituted cyclohexane ring from venlafaxine, followed by a double bond formation. The C_9_H_11_O^+^ fragment was produced from the *m*/*z* 178 ion through the loss of the -NC_2_H_5_ group. These fragmentation patterns are consistent with the hypothesized structures reported in Scheme 1 for both V276 isomers.

Consistent with the results reported by Santoke et al. [32], several hydroxylated derivatives of venlafaxine were identified, resulting from HO^•^/O_2_^•−^ radical attack on the venlafaxine molecule. Five isomers, with a molecular formula of C_17_H_28_NO_3_^+^ (*m*/*z* 294.2063), were detected at retention times ranging from 8.49 to 9.47 min, indicating monohydroxylation at various positions of the parent compound. Two of these isomers were present in trace amounts which precluded MS^n^ fragmentation for structural elucidation, and were therefore excluded from further analysis.

**Figure 6:**
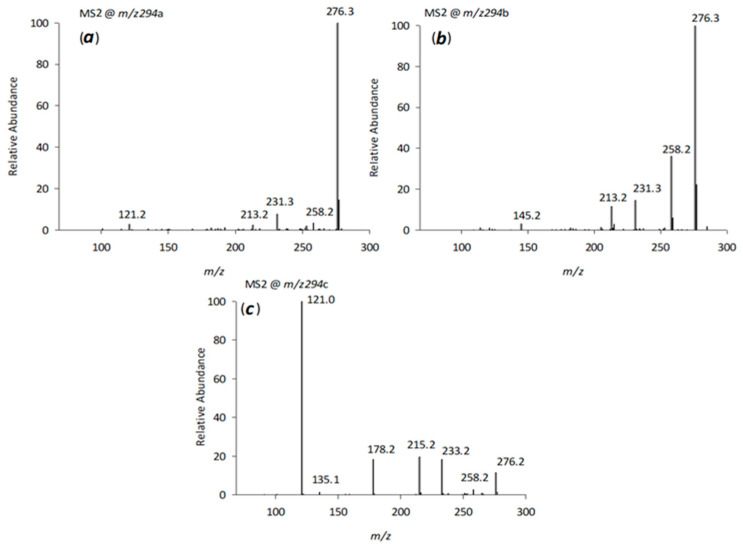
CID-MS^n^ spectra (**a**–**c**) in positive mode of the [M+H]^+^ ion of the isomers at *m*/*z* 294 generated by applying relative collision energies between 30% and 35%.

The structure of venlafaxine provides multiple potential mono-oxidation sites, which can result in products with varying polarities, including hydroxylation of the aromatic or cyclohexane ring, as well as the methylene carbon or methyl groups attached to the amine moiety. MS^n^ studies (Figure 6 a–c) revealed distinct fragmentation pathways for the three major isomers, V294a, V294b, and V294c (peaks 8, 9, and 10 in Figure 4), and allowed the proposal of possible -OH attack positions.

MS^2^ fragmentation of V294a and V294b showed the loss of H_2_O (*m*/*z* 276), a feature also observed in the venlafaxine spectrum, suggesting hydroxylation on the cyclohexane ring or the methylene group adjacent to nitrogen. MS^3^ fragmentation of the *m*/*z* 276 ion yielded a fragment at *m*/*z* 231, consistent with a modified cyclohexane ring containing an additional double bond compared to the *m*/*z* 215 fragment of venlafaxine [10]. The fragment at *m*/*z* 213 further supported the presence of this additional double bond in the cyclohexane ring. However, the precise -OH group position within the cyclohexane ring could not be determined for these two isomers.

For isomer V294c (C_17_H_28_NO_3_^+^, *m*/*z* 294.2053), its retention time (RT 9.47 min, immediately after venlafaxine), a mass difference of 16 Da compared to venlafaxine, and the elemental composition derived from accurate mass measurements suggested the formation of a product resulting from an -OH radical attack on either the methylene carbon or the aromatic ring. Fragments at *m*/*z* 276 and 215 (observed in venlafaxine) and a fragment at *m*/*z* 233 (resulting from the loss of the -NC_2_H_7_ amino group, −45 Da) were consistent with monohydroxylation of the aromatic ring, likely at the ortho- or para-position relative to the methoxyl group (Scheme 1).

Electrochemical degradation of venlafaxine may proceed through a combination of dehydrogenation and hydroxylation reactions. This hypothesis was supported by the identification of a transformation product with a molecular ion at *m*/*z* 292.1903 (peak 7 in Figure 4), consistent with the protonated molecular formula C_17_H_26_O_3_N^+^. This suggested the introduction of an oxygen atom followed by dehydrogenation. The presence of fragment ions at *m*/*z* 274, 256, and 229, which are two mass units lower than the corresponding fragments observed in V276a (*m*/*z* 276, 258, and 231), indicated the formation of an additional double bond within the cyclohexane ring. This structural feature is reflected in the proposed structure of V292 (Scheme 1).

**Figure 7:**
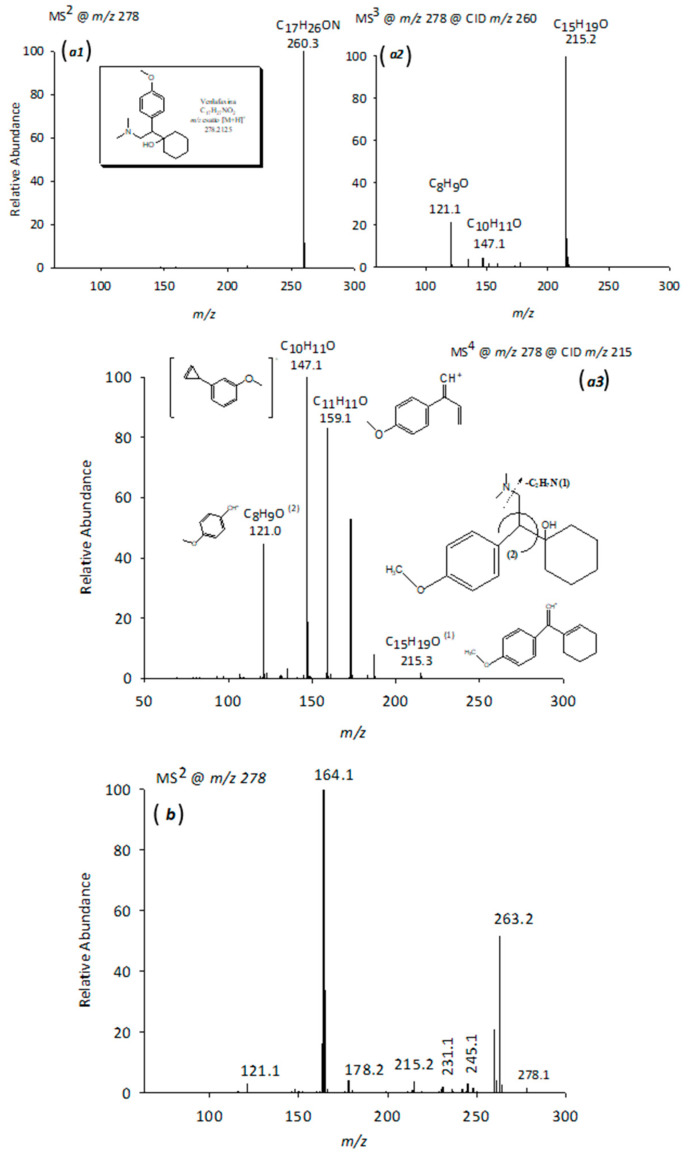
CID-MS^n^ spectra (**a1**–**a3**) in positive mode of the [M+H]^+^ ion of the VFX at *m*/*z* 278 generated by applying relative collision energies between 30% and 35% and CID-MS^2^ spectrum (**b**) in positive mode of the [M+H]^+^ ion of the compound at *m*/*z* 278 generated by applying relative collision energies between 30% and 35%.

Figure 7 displays the MS^2^-MS^4^ spectra of venlafaxine, with the main fragment ions detailed in Table 1 and their proposed structures illustrated in panels a1–a3. Figure 7b presents the MS^2^ spectrum of its isomer V278 (C_16_H_22_O_3_N^+^, peak 11 in Figure 4), which exhibited an identical nominal mass to venlafaxine but a distinct exact mass. A structural modification was proposed, wherein the nitrogen-bonded methyl group is substituted by a hydroxyl group. This hypothesis accounted for the absence of the *m*/*z* 233 fragment in the MS^2^ spectrum of V278 (Figure 7b), a fragment typically associated with dimethylamine loss (−45 Da) in the MS^2^ spectrum. Instead, a fragment at *m*/*z* 231 was observed, corresponding to a mass loss of 47 Da. This 2 Da increase was attributed to the substitution of the -CH_3_ group with a -OH group in the molecule. Furthermore, a significant fragment at *m*/*z* 263 (−15 Da), indicative of the loss of the methoxy-bound methyl group and corresponding to the C_15_H_21_O_3_N^+^ ion, was detected. This observation is consistent with previously published data [32]. The base peak at *m*/*z* 164.1 in the mass spectrum of the venlafaxine isomer may correspond to the 4-methoxyphenyl-ethyl fragment ion, resulting from the cleavage of the bond between the cyclohexanol ring and the ethyl group. The characteristic fragment at *m*/*z* 121, assigned to 1-methoxy-4-methylbenzene, was also present within the spectrum.

N-desmethylvenlafaxine, a degradation product resulting from the N-demethylation of venlafaxine, was identified in the mass chromatogram at a retention time of 8.65 min. This product exhibited an accurate *m*/*z* of 264.1958 and a molecular formula of C_16_H_26_O_2_N^+^. As detailed in Table 1, the MS^n^ fragmentation of the molecular ion yielded a fragment ion at *m*/*z* 246 (C_16_H_23_NO^+^), corresponding to a neutral loss of 18 Da (H_2_O). The presence of the recurring peak at *m*/*z* 215 (C_15_H_19_O^+^) supported the proposed structure.

Building upon the characterization of venlafaxine degradation products, this study identified V196, a compound previously reported in the electrochemical degradation processes of venlafaxine at pH 9 [30]. V196 was detected with a retention time of 4.46 min and an *m*/*z* of 196.1332.

As detailed in Table 1, MS^2^ fragmentation of the molecular ion at *m*/*z* 196 yielded a prominent peak at *m*/*z* 178, corresponding to the C_11_H_16_NO^+^ fragment. This fragment, also observed in the fragmentation of the V276a isomer, results from the loss of a water molecule (18 Da) from the precursor ion. Additionally, fragments at *m*/*z* 135 and 147, previously identified in the MS^n^ spectra of venlafaxine with established structures, were observed, further supporting the proposed structure of V196. The fragment at *m*/*z* 163 (C_10_H_13_ON^+^), attributable to N- or O-demethylation of the *m*/*z* 178 ion, was also consistent with the hypothesized structure of V196.

V194, characterized by an accurate *m*/*z* of 194.1176, was identified as a key degradation product in this study. Fragmentation of V194 yielded three distinct fragment ions at *m*/*z* 58, 121, and 149. The peak at *m*/*z* 121, also observed in the MS^4^ spectrum of venlafaxine, was attributed to the C_8_H_9_O^+^ ion. The peak at *m*/*z* 58, consistent with literature data, corresponds to the dimethyl-methylene-ammonium ion (C_3_NH_8_^+^), a characteristic fragment of venlafaxine and its degradation products, which exhibited stability during fragmentation. The fragment ion at *m*/*z* 149, matching to molecular formula C_9_H_9_O_2_^+^, arises from the loss of the dimethylamine moiety (−45 Da) from the V194 molecule. The presence of these fragment ions, particularly the one at *m*/*z* 149, provided strong support for the proposed structure of V194.

Concerning the formation pathways of these transformation products, in the degradation process studied, after 7 h of degradation, venlafaxine and the transformation products were reduced by more than 94% of the initial concentration. The formation of these transformation products occurred via specific reaction mechanisms, including demethylation, dehydration, aromatic ring hydroxylation, cyclohexane ring hydroxylation and shortening, and nitrogen group modification. Notably, electrochemical degradation at pH 9 yielded three distinct mono-hydroxylated products (V294a, b, c), identified in the degradation mixture with *m*/*z* 294.2064, resulting from aromatic and cyclohexane ring hydroxylations. These products have been previously reported only in photodegradation and advanced oxidation processes [10,32]. Other reactions led to lower molecular weight products, with V194 being the smallest. Demethylation, dehydration, and subsequent hydroxylations/oxidations constitute key stages in the transformation process.

Electrochemical degradation reactions are crucial not only for the efficacy of decontamination and removal processes but also for the generation of transformation products, which can also form in natural environments through biotic and abiotic processes. Evaluating the reliability of a pollutant degradation treatment necessitates assessing the nature of these products, as they may exhibit higher toxicity than the parent compound. Consequently, a primary objective of this study was to determine the residual toxicity of venlafaxine transformation products generated during electrochemical treatment.

### 2.4. Evaluation of the Ecotoxicity of Venlafaxine Degradation Products

The electrochemical degradation of pharmaceuticals can yield transformation products with varying toxicity compared to the parent compound [33]. Consequently, evaluating the ecotoxicity of these degradation products using in vivo, in vitro, or in silico methods is crucial. In vivo tests involve live animal studies, while in vitro assays use cell lines. However, due to the limited availability of commercial standards, these approaches are often unfeasible for assessing the ecotoxicity of pharmaceutical degradation products like venlafaxine. In silico analysis employs computational algorithms to predict substance properties, such as toxicity, based on structural and experimental data of analogous compounds.

**Figure 8:**
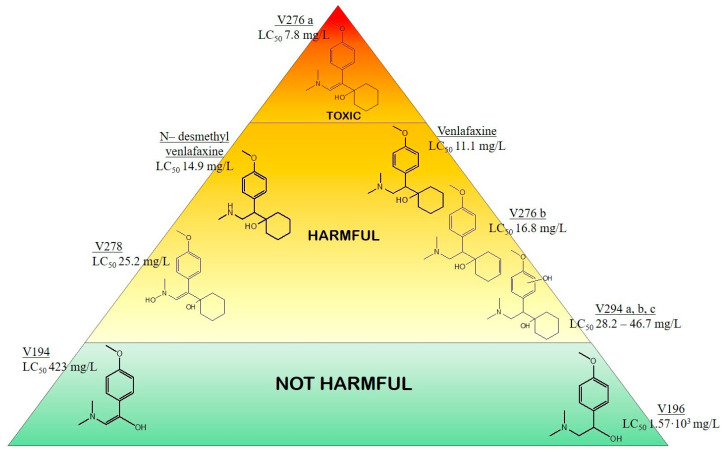
Ecotoxicity classification of venlafaxine and its degradation products and LC_50_ (mg/L) values calculated by ECOSAR software. Ranges (mg/L) and their respective characterization: 1 < LC_50_ < 10 toxic (red); 10 < LC_50_ < 100 harmful (orange/yellow); LC_50_ > 100 not harmful (green).

A well-established model is the ECOSAR (Ecological Structure Activity Relationship) software [34]. ECOSAR estimates the toxicity of parent and transformation products by calculating the LC_50_, defined as the median lethal concentration (mg/L) causing 50% mortality in test organisms. Using ECOSAR, the ecotoxicity of venlafaxine degradation products, which had been identified by LC-MS and LC-MS^n^, was evaluated. Chemical structures of venlafaxine and its major degradation products were created with ChemDraw, Ultra 12.0 and LC_50_ values for Daphnia magna were then calculated. The resulting LC_50_ values, alongside the chemical structures, are presented in Figure 8 using a tiered pyramid format.

The tiered pyramid effectively illustrates the relative ecotoxicity of venlafaxine and its major degradation products, as determined by LC_50_ values. A comparative analysis of molecular structures showed a predominant trend: electrochemical degradation of venlafaxine forms products with reduced toxicity compared to the parent compound.

Venlafaxine (LC_50_ 11.1 mg/L), with a complex bicyclic structure and a tertiary amine group, exhibited moderate toxicity, likely due to structural features that facilitate interactions with biological targets. The degradation process generally yielded compounds with diminished ecotoxicity. However, V276a (LC_50_ 7.8 mg/L) stands out as an exception since exhibited slightly higher toxicity than the parent venlafaxine. Specifically, compounds V194 (LC_50_ 423 mg/L) and V196 (LC_50_ 1.57 × 10^3^ mg/L) demonstrated the most significant toxicity reduction. This was attributed to their simplified aromatic structures and increased oxidation, which increased polarity and water solubility. As a result, the potential for bioaccumulation was reduced. The difference in toxicity between V194 and V196, highlighted by the LC_50_ increase upon double bond saturation (V194 to V196), indicated that the presence of the double bond significantly contributes to higher toxicity in lower molecular weight products.

Other degradation products, such as N-desmethyl venlafaxine (LC_50_ 14.9 mg/L), V276b (LC_50_ 16.8 mg/L), V278 (LC_50_ 25.2 mg/L), and V294 a, b, c (LC_50_ 28.2–46.7 mg/L), also showed lower toxicity than venlafaxine, likely due to increased hydroxylation and structural degradation, which enhances polarity and facilitates compound elimination.

The predominance of less toxic compounds, resulting from increased oxidation, hydroxylation, and structural simplification, highlights the effectiveness of electrochemical degradation in reducing venlafaxine’s environmental impact. Consistent with established environmental toxicology principles, where increased polarity and structural simplification minimize organic compound ecotoxicity, this study’s observation aligns with previous research, such as Voigt et al. [16], showing that increased hydroxyl group substituents decrease ecotoxicity. This agreement underscores the method’s potential for sustainable pharmaceutical waste management.

### 2.5. Electrochemical Degradation of Venlafaxine: Advantages of Platinum Electrodes

The research activities that led to the development of the electrochemical degradation method for VFX using platinum electrodes are outlined in their key stages in Scheme S1 of the Supporting Materials. As detailed in our study, this method offers several important advantages over alternative approaches, making a compelling case for its adoption (Table 2).

A critical consideration in electrochemical applications is electrode performance, specifically operational longevity and ease of maintenance. Pt electrodes, employed in our study with 0.1 M Na_2_SO_4_ electrolyte at pH 9 and a current density of 25 mA/cm^2^, exhibited remarkable stability and a straightforward cleaning protocol. This contrasts with BDD electrodes, which, while widely explored (e.g., in [15,16,17,18,35]), can present challenges related to their specific properties and maintenance requirements. The inherent robustness of Pt electrodes contributed to enhanced experimental reproducibility and a potentially prolonged operational lifetime, reducing both procedural complexity and long-term costs.

A significant advantage of the Pt electrode-mediated approach, as implemented in our study, was the utilization of a chloride-free electrolyte (0.1 M Na_2_SO_4_). This is particularly important when compared to studies such as [17,18], where the inclusion of chloride ions (e.g., 0.02 M NaCl), while potentially accelerating degradation kinetics, introduces the risk of forming hazardous halogenated byproducts. The absence of chloride in the Pt-based methodology aligns with the imperative for environmentally benign remediation strategies.

Among the compared studies, as reported in Table 2, only study [18] did not identify degradation intermediates. Zhu et al. [17] utilized GC-MS and reported different intermediates compared to this work and the study by Voigt et al. [16], which employed LC-HRMS. Voigt et al. [16] identified four intermediates, while our study revealed ten. Study [15] also reported four intermediates identified by LC-HRMS, two of which were chlorinated due to the use of chloride electrolyte. These findings strongly suggest that the choice of electrode material significantly influences the formation of degradation intermediates.

A hallmark of responsible environmental research is a thorough evaluation of potential ecological impacts. While our study incorporates an in silico ecotoxicity assessment using ECOSAR, it is essential to acknowledge that only two other investigations address this aspect. For instance, study [16] employs in silico QSAR analysis, revealing four intermediates for the electrochemical degradation process, and study [17] includes toxicity tests on *Chlorella vulgaris*. This comprehensive analysis, although implemented with different methodologies, provides critical insights into the environmental fate and potential risks associated with the degradation process.

It is acknowledged that the extended degradation times might be considered a limitation. However, this potential drawback is effectively counterbalanced by the enhanced stability, safety profile regarding byproduct formation, and overall sustainability of the Pt electrode-mediated process. Furthermore, in our study, we calculated that the energy consumption for achieving 94% degradation of 25 ppm VFX was 98 kWh/m^3^. Energy consumption is widely recognized as a key parameter in evaluating the practical implementation of EAOPs; nevertheless, none of the studies summarized in the table included its estimation.

Briefly, the electrochemical degradation of VFX using Pt electrodes represents a viable and sustainable remediation strategy. It is characterized by distinct advantages in operational stability, environmental safety, and comprehensive ecotoxicological assessment.

## 3. Materials and Methods

### 3.1. Materials

Acetonitrile (99.9%), nitric acid (70%), sodium hydroxide (98%), sodium phosphate monobasic (99%), sodium phosphate dibasic (99%), sodium sulfate (99%), ethanol (99.9%), methanol (99.8%), and venlafaxine chloridrate (Pharmaceutical Secondary Standard, certified reference material) were purchased from Sigma-Aldrich (Steinheim, Germany). Powdered alumina was supplied by Buehler (Micropolish II 0.05 µm, deagglomerated gamma alumina). Ultra-pure water used in all the experiments was obtained from a combined Elix5/Milli-Q system (Millipore, S.p.A., Milan, Italy). Platinum foil (99.9%) and graphite plate were obtained from Sigma-Aldrich (Steinheim, Germany). Glassy carbon plates were purchased from HTW Hochtemperatur Werkstoffe GmbH (Thierhaupten, Germany).

### 3.2. Electrochemical Experiments

#### 3.2.1. Cyclic Voltammetry and Differential Pulse Voltammetry

A 263A potentiostat/galvanostat (EG&G Princeton Applied Research, Princeton, NJ, USA) was employed to carry out cyclic voltammetry experiments. Data were acquired by using the M270 software version 4.23 (EG&G). A conventional three-electrode cell was used consisting of a saturated calomel reference electrode (SCE), a platinum counter electrode, and a working electrode made of a glassy carbon disk or a platinum disk inserted in a PTFE body. DPV experiments were carried out with a CHI660B potentiostat (Shanghai CH Instrument Company, Shanghai, China).

The GC surface was cleaned following an optimized procedure [36]. The electrode was mechanically polished with abrasion by alumina (0.05 µm particles) and sequentially sonicated firstly in double distilled water and ethanol (*v*/*v* 1:1) for 2 min, then in double distilled water and nitric acid (*v*/*v* 1:1) for 2 min and finally in double distilled water for 2 min at the end of each sonication, the electrode was rinsed with pure water.

Platinum surface cleaning was performed as follows [24]: the electrode was mechanically polished by abrasion with alumina (0.05 µm particles) and sonicated in double distilled water; then, it was sonicated for a few minutes in hot nitric acid (70%) for chemical cleaning; finally, it was electrochemically treated by cycling in 0.5 M sulfuric acid in the potential range of −0.225 V/+1.25 V (vs. SCE) at a scan rate of 100 mV/s for the number of cycles sufficient to get a steady state current profile.

#### 3.2.2. Galvanostatic Electrolysis

The VFX degradation by galvanostatic electrolysis was carried out employing a 263A potentiostat/galvanostat (EG&G Princeton Applied Research, Princeton, NJ, USA). A two electrodes configuration was adopted: the graphite plate (3 mm × 20 mm × 60 mm) was used as the cathode whereas the platinum foil (0.127 × 25 × 25 mm) or the glassy carbon plate (1 mm × 20 mm × 40 mm) were used as anodes. The different anodes were immersed in the electrolytic solution made of sodium sulfate 0.1 M at pH 9 (unless otherwise stated) by exposing the same surface area (8 cm^2^). The distance between anode and cathode in the solution was 2 cm. The volume of the reactor was 100 mL. The solution was kept at room temperature under stirring during the experiment.

### 3.3. LC-UV Conditions

The LC-UV experiments were performed on an Agilent 1200 series gradient HPLC system (Agilent Technologies, Santa Clara, CA, USA) equipped with a quaternary gradient pump, a diode array detector (DAD, 190–950 nm), and a standard autosampler (0.1 µL–100 µL). A C_18_ Supelcosil LC-ABZ column (25 cm × 3 mm, 5 μm) from Supelco (Supelco Inc., Bellefonte, PA, USA) was employed at room temperature. The mobile phase consisted of ultrapure water (A) and acetonitrile (B), with the following gradient elution program: 0 min, 90% B; 3 min, 80% B; 4–8 min, 70% B; 9 min, 80% B; 10–12 min, 90% B. The flow rate was 0.8 mL/min, with an injection volume of 20 µL; chromatograms were acquired at 226 nm, and UV–Vis spectra were recorded in the 190–400 nm range.

### 3.4. LC-MS/MS Analysis

The LC-MS/MS analysis of the degraded VFX solutions was carried out by using an HPLC Accela AS system (Thermo Fisher Scientific, Bremen, Germany) coupled to a LIT-Orbitrap XL (Thermo Fisher Scientific, Bremen, Germany), with an ESI interface operating in positive ionization mode. The optimal values of the source parameters were as follows: spray voltage of 4.27 kV, capillary temperature of 280 °C, and sheath gas flow rate of 80 AU (arbitrary units). The compound identification and fragmentation studies were carried out by setting full scan (in a range of *m*/*z* 50–500) and data-dependent scan (DDA) acquisition modes. For the chromatographic separation, the same chromatographic column, mobile phase, and elution gradient used in the LC-UV experiments were employed at a flow rate of 0.8 mL/min by splitting 3:1 post-column. Low-resolution MS^n^ experiments were performed by collisional induced dissociation (CID), employing a collisional energy between 30% and 35%. Data obtained were processed using Xcalibur version 2.0 software. Mass spectra were imported, elaborated, and plotted by SigmaPlot 10.0 (Systat Software, Inc., London, UK). Structures of identified molecules and moieties were drawn by ChemDraw Ultra 12.0 (CambridgeSoft Corporation, Cambridge, MA, USA).

### 3.5. In Silico Toxicity Evaluation

The in silico toxicological evaluation of venlafaxine and its degradation products was performed using the ECOSAR program based on the chemical structure/toxicity relationship. The degree of acute toxicity is estimated on the LC_50_, on daphnids after 48 h of contact [37]. The ECOSAR package, available for free download from the US EPA website [38], categorizes input compounds into one or more chemical classes. It then applies a hydrophobicity-based structure–activity relationship (SAR) relevant to the class(es) to predict ecotoxicity. The logarithm of the octanol:water partition coefficient is used as the input parameter. The SARs establish a correlation between the physicochemical properties of chemicals and their aquatic toxicity. The models incorporate more than 150 SARs, covering over 50 chemical classes. The underlying assumption is that the aquatic toxicity of a compound can be predicted based on known values for similar compounds within the same class.

## 4. Conclusions

This study demonstrated the potential of advanced electrochemical oxidation processes based on galvanostatic electrolysis for purifying water from venlafaxine, a drug recently included on the EU Watch List due to its widespread occurrence and persistence. A platinum electrode was found to be suitable for this purpose and proved to be a good alternative to the commonly used BDD electrode. It offers the advantage of a well-established cleaning procedure, ensuring high reproducibility of its surface state, and is stable and durable over extended periods of use. The electrochemical degradation of VFX occurred through indirect oxidation by highly reactive species, specifically hydroxyl and sulfate radicals, which were electrochemically generated on the Pt anode during electrolysis.

An HPLC-UV method was used to derive the degradation curve of VFX. The experimental conditions for electrolysis regarding electrolyte pH and current density were optimized. A higher degradation yield of VFX was observed in a 0.1 M Na_2_SO_4_ solution at pH 9, applying a current density of 25 mA/cm^2^. Additionally, a concentration-dependent degradation efficiency was observed, with higher efficiency at lower VFX concentrations, consistent with the first-order kinetic derived. Over longer time scales, after 7 h of electrolysis, VFX degradation was found to be satisfactory (94%), even at a concentration of 25 ppm.

This study focused on identifying the transformation intermediates of venlafaxine through LC-ESI-MS^n^ and evaluating their environmental impact. Transformation products are formed mainly through demethylation, dehydration, and hydroxylation/shortening of aromatic and cyclohexane rings. In particular, the hydroxylation reactions of the aromatic and cyclohexane rings led to three different monohydroxylation products, which were previously identified only for photodegradation processes. Other reactions resulted in lower molecular weight products, including N-desmethylvenlafaxine and two isomers of the V276 product.

Finally, an in silico ecotoxicity assessment using ECOSAR demonstrated that almost all intermediates were less toxic than VFX. Furthermore, the most abundant degradation product (V194) exhibited the lowest toxicity. Consequently, the proposed electrochemical degradation method for venlafaxine removal from contaminated water can be considered environmentally sustainable.

## Figures and Tables

**Table 1:** Chromatographic and mass spectrometry data for the main transformation products resulting from the electrochemical degradation of venlafaxine, identified by LC-ESI(+)-CID-MS^n^.

N.	Compounds	Retention Time (min)	Molecular Formula	^1^ [M+H]^+^ *m*/*z* Accurate	Error (ppm)	Main Fragments [M+H]^+^ *m*/*z* Nominal
1	VFX	9.24	C_17_H_27_O_2_N	278.2111	−1.42	260; 215;159; 147; 121; 58
2	V276a	8.80	C_17_H_25_O_2_N	276.1954	−1.47	258; 246; 213; 178; 145; 135; 121
3	V276b	9.48	C_17_H_25_O_2_N	276.1956	−0.89	258; 246; 215; 159; 147; 121
4	V264 (N-desmethylVFX)	8.65	C_16_H_25_O_2_N	264.1956	−0.70	246; 215; 121
5	V196	4.46	C_11_H_17_O_2_N	196.1333	0.28	178; 163; 147; 135
6	V194	5.19	C_11_H_15_O_2_N	194.1176	0.28	149; 121; 58
7	V292	8.28	C_17_H_25_O_3_N	292.1903	−1.40	274; 256; 121
8	V294a	8.49	C_17_H_27_O_3_N	294.2061	−1.09	276; 258; 231; 213; 121
9	V294b	8.83	C_17_H_27_O_3_N	294.2059	−1.60	276; 258; 231; 213; 145
10	V294c	9.47	C_17_H_27_O_3_N	294.2060	−1.30	276; 258; 233; 215; 178; 135; 121
11	V278	8.22	C_16_H_23_O_3_N	278.1749	−0.72	263; 245; 231; 178; 164; 121

^1^ average value of n = 3 *m*/*z* measurements.

**Table 2:** Operational parameters and main outputs of different EAOP methods for VFX degradation.

Electrode Material	Electrolyte Composition	pH	Current Density	Initial VFX Concentration	Degradation Efficiency	Identified Transformation Products	Toxicity Data	References
Pt	0.1 M Na_2_SO_4_	9	25 mA/cm^2^	25 mg/L	94% (7 h)	10 by-products identified by LC-MS/MS and HRMS	in silico (ECOSAR)	This study
BDD	HCl	3	not reported	20 mg/L	not detailed (deduced value of ~100% after around 3 h)	4 by-products identified by LC-HRMS	in silico (QSAR)	[16]
BDD	0.1 M Na_2_SO_4_ 0.02 M NaCl	6.5	100 mA/cm^2^	25 mg/L	98% (5 min)	16 by-products (electrolyte NaCl) + 6 by-products (electrolyte Na_2_SO_4_) identified by GC-MS	toxicity test on *Chlorella Vulgaris*	[17]
BDD	0.1 M Na_2_SO_4_ 0.02 M NaCl	6.5	100 mA/cm^2^	25 mg/L	98.5% (5 min)	not evaluated	not evaluated	[18]
BDD	waste water (specific electrical conductivity 1455 μS/cm)	7.2	50 mA/cm^2^	1 mg/L	99.9% (240 min)	3 by-products (2 of which chlorinated) identified by LC-MS/MS and HRMS	not evaluated	[15]

## Data Availability

Data are available on request due to privacy restrictions.

## Supplementary Materials

The following supporting information can be downloaded at https://www.mdpi.com/article/10.3390/molecules30091881/s1, Figure S1: Differential pulse voltammograms acquired on a conventional glassy carbon electrode for a 25 ppm VFX solution in phosphate buffer 0.1 M at pH 7 before (black curve) and after (red curve) the galvanostatic electrolysis carried out by employing glassy carbon (a) and platinum (b) as anode. A current density of 10 mA/cm^2^ was applied for 60 minutes; Figure S2: Differential pulse voltammograms acquired on a conventional glassy carbon electrode for a 25 ppm VFX solution in phosphate buffer 0.1 M at pH 7 containing 200 mM ethanol before (black curve) and after (red curve) the galvanostatic electrolysis carried out by employing glassy carbon as anode. A current density of 10 mA/cm^2^ was applied for 60 minutes; Scheme S1: Representation of the key stages of the research activity that enabled the development of the electrochemical degradation method for venlafaxine.
