# Supplementary material for: Electrochemical Degradation of Venlafaxine on Platinum Electrodes: Identification of Transformation Products by LC-MS/MS and In Silico Ecotoxicity Assessment
Source: Molecules. 2025 Apr 23;30(9):1881. doi: 10.3390/molecules30091881 (PMC12073582; doi:10.3390/molecules30091881)
Supplement: Supplementary file 1 [file molecules-30-01881-s001.zip › molecules-3570405-supplementary.pdf]

molecules

Supplementary Materials

Electrochemical degradation of venlafaxine on platinum electrodes: identification of transformation products by LC-MS/MS and *in silico* ecotoxicity assessment

Angelica Zizzamia[1], Veronica Pasquariello[1], Filomena Lelario[1,*], Carmen Tesoro and Rosanna Ciriello[1,*]

[1] Department of Basic and Applied Sciences, University of Basilicata, 85100 Potenza, Italy
* Correspondence: F.L. filomena.lelario@unibas.it; R.C. rosanna.ciriello@unibas.it

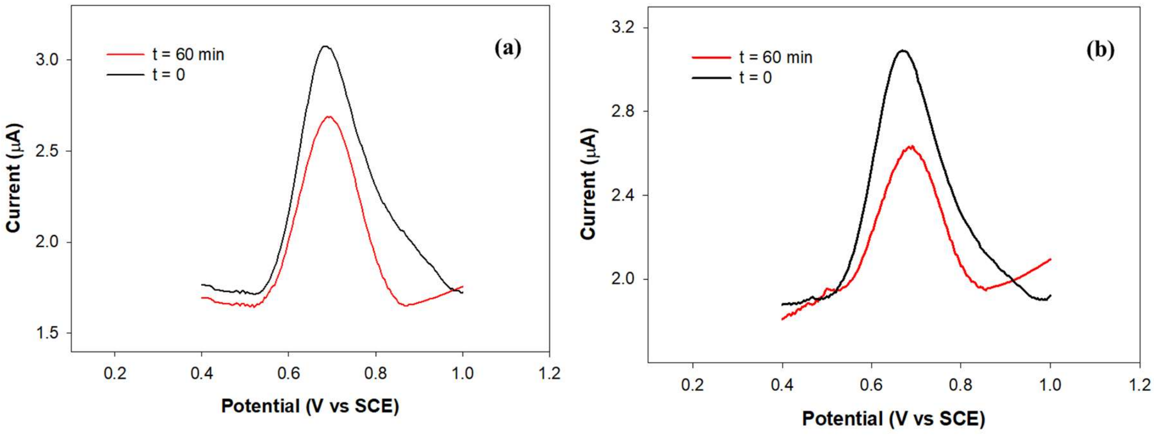

Figure S1. Differential pulse voltammograms acquired on a conventional glassy carbon electrode for a 25 ppm VFX solution in phosphate buffer 0.1 M at pH 7 before (black curve) and after (red curve) the galvanostatic electrolysis carried out by employing glassy carbon (a) and platinum (b) as anode. A current density of 10 mA/cm² was applied for 60 minutes.

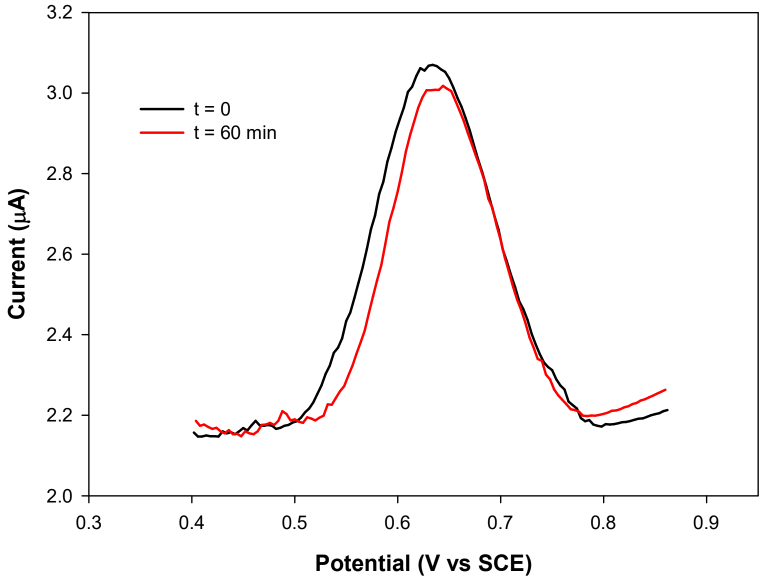

Figure S2. Differential pulse voltammograms acquired on a conventional glassy carbon electrode for a 25 ppm VFX solution in phosphate buffer 0.1 M at pH 7 containing 200 mM ethanol before (black curve) and after (red curve) the galvanostatic electrolysis carried out by employing glassy carbon as anode. A current density of 10 mA/cm² was applied for 60 minutes.

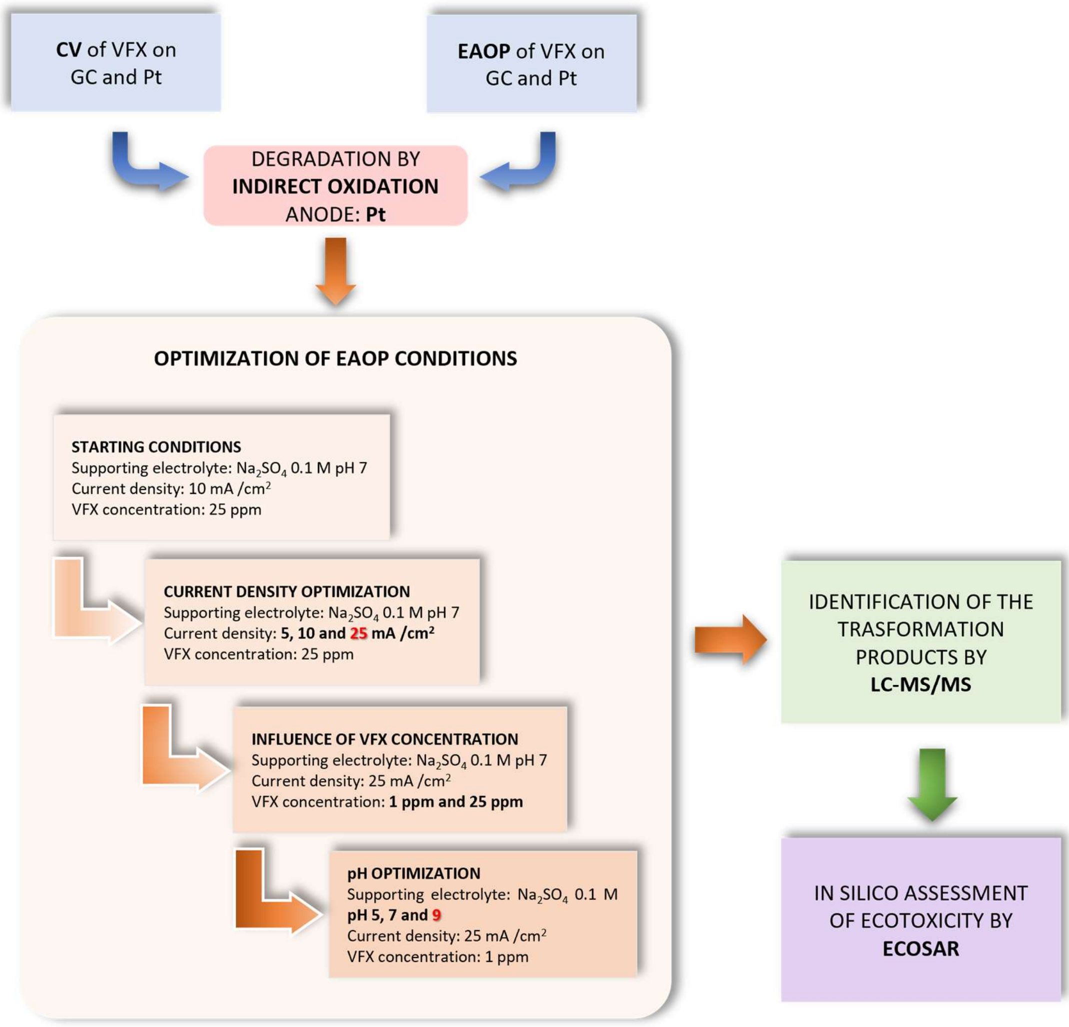

Scheme S1- Representation of the key stages of the research activity that enabled the development of the electrochemical degradation method for venlafaxine
